# Development of a question prompt list to support Consent for Genomic Testing (CoGenT) and research

**DOI:** 10.1007/s00439-026-02839-0

**Published:** 2026-05-21

**Authors:** Jolyn Hersch, Lauren O’Hara, Phyllis Butow, Mandy Ballinger, Rebekah Laidsaar-Powell, Nicci Bartley, Kirsten McCaffery, Anastasia Latin, Christine Cockburn, Caitlin Delaney, Katherine M. Tucker, Ilona Juraskova

**Affiliations:** 1 Psycho-Oncology Co-operative Research Group (PoCoG), School of Psychology, Faculty of Science, The University of Sydney, Sydney, Australia; 2 Sydney Health Literacy Lab, School of Public Health, Faculty of Medicine and Health, The University of Sydney, Sydney, Australia; 3 Centre for Molecular Oncology, School of Biomedical Sciences, Faculty of Medicine and Health, University of New South Wales, Sydney, Australia; 4 Rare Cancers Australia, Bowral, Australia; 5 CareFully, Sydney, Australia; 6 Hereditary Cancer Centre, Nelune Comprehensive Cancer Centre, Prince of Wales Hospital, Sydney, Australia; 7 School of Clinical Medicine, Faculty of Medicine and Health, University of New South Wales, Sydney, Australia

## Abstract

While genomic testing is increasingly utilised to personalise cancer treatment, its complexity creates challenges in achieving informed consent. Partnering with patients, carers, researchers and health professionals, we developed a Question Prompt List (QPL) aiming to support communication and informed consent for tumour profiling and genomic research in oncology. Participants (*n* = 38) included cancer patients participating (or not) in genomic research, carers, researchers and clinicians. Two rounds of interviews elicited information needs and suggested questions, followed by feedback on the draft QPL and its potential value. We produced concise answers for all questions, edited wording using health literacy principles, and refined according to feedback. The QPL includes 29 items arranged into three sections: Comprehensive Genomic Profiling (CGP), Results of CGP, and About the Genomic Research Study. Participants believed the QPL would empower patients and carers, support clinicians and researchers to engage with patient/family queries, and enable better informed consent in genomic oncology. The Consent in Genomic Testing (CoGenT) Question Prompt List, including evidence-based answers, has been developed rigorously and collaboratively. This simple and flexible tool is perceived as acceptable and useful for patients, carers, clinicians, and research personnel engaging in informed consent for genomic research including tumour profiling.

**Supplementary Information:**

The online version contains supplementary material available at 10.1007/s00439-026-02839-0.

Genomic testing, involving sequencing of DNA from tumour tissue, has potential to transform cancer management and outcomes. By identifying deleterious variants in tumours, comprehensive genomic profiling (CGP) facilitates personalised therapy (Seed 2021). However, CGP may generate information not only relevant to the diagnosed cancer but also other conditions or of unknown significance (Best et al. 2020). Due to the complexity and potential outcomes of CGP, which patients often access by participating in research, there are concerns about patients’ ability to comprehend the information needed for informed consent (Nikitara et al. 2025).

Research shows some patients have poor knowledge and feel uncertain about CGP, its utility, and the implications of results (Best et al. 2019; Bartley et al. 2020). They may overestimate the likelihood of results informing treatment (Davies et al. 2020), particularly when treatment options have been exhausted (Butow et al. 2020), or may not consider potential implications such as incidental germline results that are significant for relatives (Best et al. 2019; Biesecker et al. 2019). Poor understanding can lead to distress when uninformative or potentially germline results are received (Best et al. 2019, 2020). Conversely, uncertainty about implications of testing (e.g., for employment and privacy) may deter patients from undergoing CGP (Bartley et al. 2020).

There is a well-established need for tailored information about CGP and open patient-clinician communication, acknowledging the substantial cognitive burden of complex information, particularly for patients with lower education or those navigating an unfamiliar healthcare system in their second language (Davies et al. 2020; Nikitara et al. 2025). Technical language and medical jargon used during both the consent process and when receiving results can hinder understanding. Patients interviewed after receiving CGP results reported that simpler, less technical language would help reduce their uncertainty (Bartley et al. 2022). Importantly, evidence suggests that patients’ self-efficacy in coping with CGP results, assessed before receiving results, predicts their mental health in subsequent months (Vatter et al. 2022). To foster autonomy, understanding, and self-efficacy, patients need support to identify their information needs, access clear information with time to digest and reflect, and communicate effectively with researchers and clinicians. One evidence-based tool that could provide such support is the question prompt list (QPL).

A QPL is a structured list of questions, typically given to patients before a healthcare consultation, aiming to ‘activate’ and empower patients and thereby improve communication and decision-making (Keinki et al. 2021). QPLs are designed to foster individualised information exchange between patients and clinicians, allowing patients to share their thoughts and concerns, and gather information tailored to their needs. Systematic reviews show that QPLs are effective in oncology settings, enhancing patient-clinician communication and increasing question asking, particularly about more difficult and sensitive topics such as prognosis or financial implications (Butow et al. 1994; Miller and Rogers 2018). QPLs can also improve cognitive (e.g., recall of information) and psychological outcomes (e.g., anxiety at follow-up) (Brandes et al. 2015). Within the tumour genomics context, a QPL has been developed to support treatment discussions for breast cancer patients who have already undergone gene expression profile testing to assess risk of recurrence, with preliminary non-randomised evaluation indicating its acceptability and potential efficacy (Jayasekera et al. 2020). However, to our knowledge no QPL currently exists to guide communication about decisions to undergo CGP or participate in research involving CGP.

To support informed consent in this complex space, we rigorously developed the Consent in Genomic Testing (CoGenT) QPL, and accompanying evidence-based answers, about undergoing CGP in a research setting. Given the importance of interest-holder involvement in QPL development (Schulte-Vieting et al. 2022), we sought input from relevant health and research professionals, cancer patients with and without CGP experience, and their carers/families to: (i) identify patient information needs when considering CGP in a research context; (ii) co-develop a QPL to support communication, decision-making, and informed consent; and (iii) obtain feedback on the QPL’s content, feasibility and helpfulness.

## Methods

### Design and procedure

The study team comprised multi-disciplinary experts in health psychology/psycho-oncology, cancer genetic counselling, health literacy/communication, patient advocacy and lived experience of advanced cancer. Two rounds of semi-structured online interviews were conducted by JH, LO and AL between August 2022 and July 2023 with patients, carers, and professionals as detailed below: the first interview elicited information needs and suggestions for questions to include in the QPL; the second sought feedback on the draft QPL. Participants received a $25 gift card for their time and input. The project was approved by the St Vincent’s Hospital Human Research Ethics Committee (2022/ETH00591).

### Participants

Eligible participants were at least 18 years old, with sufficient English proficiency and ability to provide informed consent. Four key groups were purposively targeted to capture diverse experiences and perspectives on appropriate CGP consent information and processes:

1. ***Cancer patients who had undergone CGP in a research context***.

Patients were recruited from the Molecular Screening and Therapeutics (MoST) study (Thavaneswaran et al. 2018), a cancer genomics research program coordinated by Omico. The MoST cohort included adults with advanced or metastatic solid cancers, primarily rare cancers, enrolled during or after their last line of effective therapy (Thavaneswaran et al. 2018). After referral to MoST by the treating clinician, patients had a consent discussion with Omico staff before undertaking tumour CGP to identify actionable molecular changes of therapeutic relevance. Omico staff emailed QPL study information to eligible MoST participants requesting expressions of interest to be contacted about the study; contact details for those interested were provided to the QPL research team.

2. ***Cancer patients who had*** ***not*** ***undergone CGP in a research context***.

Patients with rare and less common cancers who had *not* undergone CGP in a research study were recruited via the charity Rare Cancers Australia (RCA) (Rare Cancers Australia). Although naïve to the research-based CGP consent process, this cohort was selected because its members were expected to have otherwise similar experiences and attributes to those in the MoST cohort. RCA invited expressions of interest by emailing members of its patient database and provided contact details for those interested to the research team.

3. ***Family members/Carers.***

Participating patients in both groups were asked to nominate to the research team a family member or carer who may also wish to participate in a separate interview.

4. ***Healthcare and Research Professionals (HCPs)***.

Eligible HCPs had experience in cancer genomics study consent processes (e.g., facilitating patient consent for MoST) or in relevant clinical work (e.g., medical oncology, clinical genetics). HCPs were identified through the research team’s professional networks, including Omico, and via snowballing.

### Consent, data collection and analysis

The researchers sent study invitations by email to the individuals identified above. Participants completed online consent and a baseline survey capturing demographics (e.g., age, education, geographic location) and cohort-specific information, including cancer information for patient groups, carer-patient relationship, and HCPs’ professional roles. Researchers then contacted participants to arrange interviews.

#### Interview 1: development stage

The semi-structured interview guide was informed by clinical trials QPL literature (Brown et al. 2011) and genomics-related psycho-oncology research (Best et al. 2019; Davies et al. 2020). Interviews elicited perceptions on what information was important and what questions would be helpful during the consent process (Table 1). Open-ended questions were followed by prompts about particular topics. Wording was tailored for each participant subgroup: respondents with experience in genomic research drew on personal or professional insights, while others, without such experience, were provided a brief description of CGP/MoST and asked to consider questions they would want to ask if (hypothetically) invited to participate. Participants were also asked to reflect on how and when a QPL might be useful during consent. Interviews were recorded and transcribed verbatim using Trint automated software.

**Table 1:** Outline of Interview Topics/Prompts—Development Stage

*Genomic Testing (CGP) in a Research Program (e.g., MoST)*
What patients are interested in finding out about genomic testing research
Questions patients may wonder but not ask (e.g. due to discomfort, lack of opportunity)
Questions arising after patients join genomic research (not covered during consent)
What patients want to know to feel confident about the research and the researchers
The most important things to ask/tell when someone is deciding whether to take part
Any other questions that would be useful to ask before joining this kind of research
Perceived importance of specific topics (prompts based on literature)
*QPL*
Perceived helpfulness of a QPL about taking part in research
Optimal timing for giving patients a QPL
Patients’ comfort asking questions, and potential supports/facilitators

Interviews underwent conventional content analysis (Hsieh and Shannon 2005) to identify information needs, determine key questions for inclusion in the QPL, and capture broader participant perspectives to guide tool development. Information needs and suggested topics relevant to informed consent were extracted from the interviews and collated into a document, retaining participants’ wording and recording how many participants mentioned or endorsed each element. Through discussion among members of the research team (JH, LO, PB and IJ), these elements were categorised, organised into a meaningful structure, and refined into a draft set of questions. There was no predetermined number of items; rather, the aim was to comprehensively represent the range of elements identified as pertinent to the intended scope.

Draft answers were developed with input from the multi-disciplinary research team and in consultation with two experts in cancer genetics and medical oncology. The team reviewed and discussed the draft’s comprehensiveness, clarity, and appropriateness, leading to further refinement. To improve readability and accessibility, all wording was assessed and edited using the SHeLL Health Literacy Editor (Ayre et al. 2024), an online tool evaluating linguistic complexity and overall text readability which recommends aiming for a Grade 8 reading level.

#### Interview 2: feedback stage

Participants in the first round of interviews were invited to take part in the second round unless health reasons prevented this. In the second round of interviews, participants reviewed the draft list of questions and answers via screen share and were invited to share their first impressions, along with any feedback, criticisms, or suggestions. Probing questions then elicited specific feedback on structure, scope, content, wording, perceived usability, and potential implementation barriers. Healthcare and research professionals were also asked to comment on the accuracy of answers and to suggest revisions where appropriate. Feedback was compiled by LO and AL and is summarised descriptively below.

**Fig. 1:**
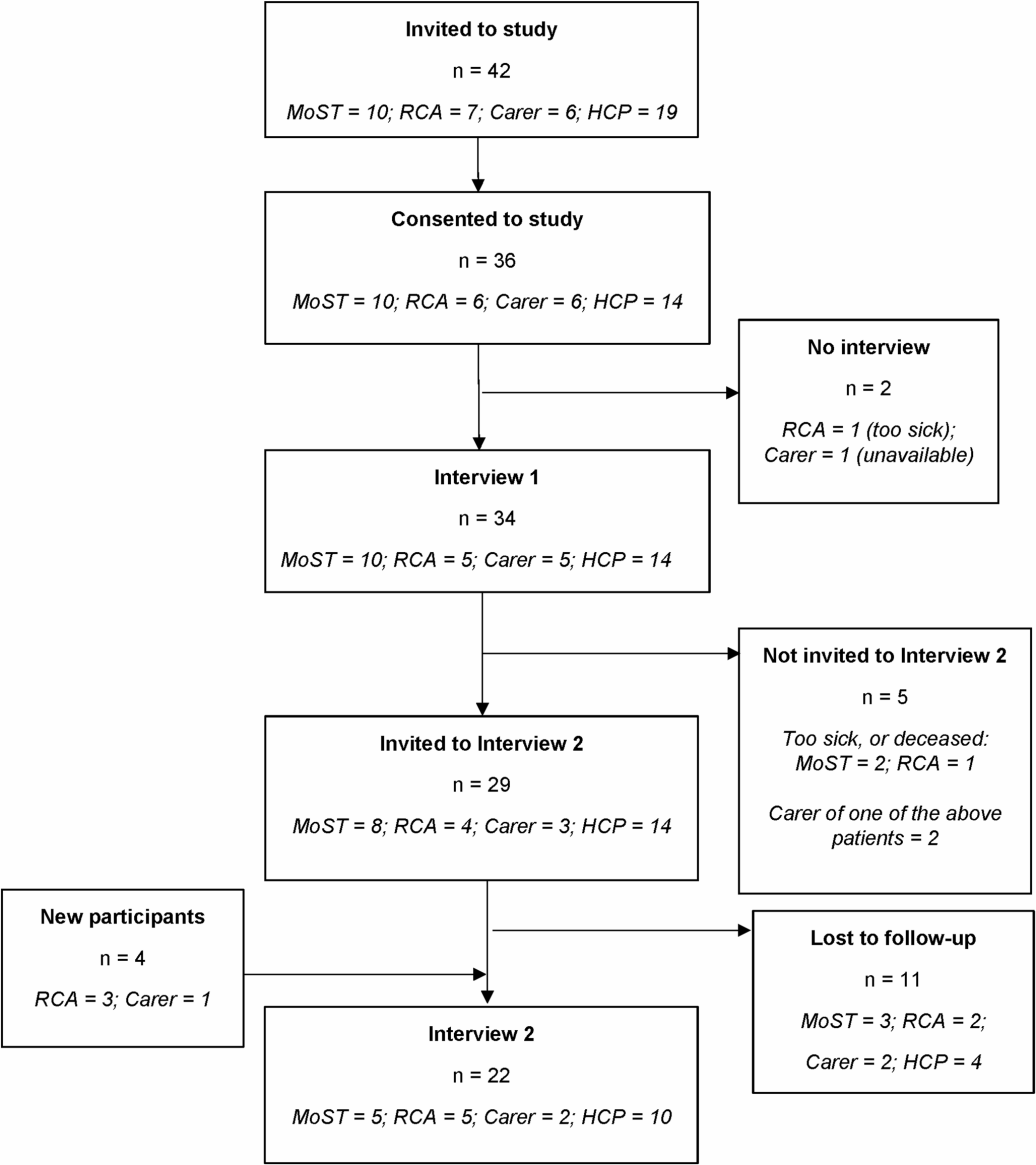
Recruitment and Participation Flowchart. *MoST:* patient from MoST genomics study,* RCA:* patient from Rare Cancers Australia,* Carer:* carer or family member of patient in this study, *HCP:* healthcare or research professional.

## Results

### Participant characteristics

Figure 1 outlines participant flow. The first interview round involved 34 participants (10 MoST participants, 5 RCA members, 5 carers/family members, and 14 HCPs). In the second round, 4 new participants joined (3 from RCA, 1 carer), while 5 participants were not re-invited based on Omico/RCA advice (3 patients due to death or severe illness, and 2 of their carers). MoST participants had been enrolled in the program up to 3 years ago, most (60%) within the past year. Among carers, 4 were spouses, 1 was the daughter and 1 a sibling of patients from MoST (*n* = 4) and RCA (*n* = 2) cohorts. Table 2 shows other characteristics.

**Table 2:** Participant Characteristics (Overall and by Subsample)

	Overall *N* = 38	MoST pts *n* = 10	RCA pts *n* = 8	Carers *n* = 6	HCPs *n* = 14
Age, M (SD) [Range]	49.0 (13.7) [26–71]	58.9 (10.7) [45–71]	51.1 (10.2) [32–68]	55.3 (10.4) [40–68]	38.1 (11.6) [26–65]
*Gender*					
Man	13	6	3	1	3
Woman	25	4	5	5	11
*Language spoken at home*					
English only	33	10	7	6	10
Other	5	0	1	0	4
*Highest level of education*					
Year 10 or below	1	1	0	0	0
Year 12	2	1	0	0	0
Vocational certificate /diploma	3	2	1	1	0
University degree	11	1	2	5	3
Postgraduate degree	21	5	5	0	11
*Location (state)*					
New South Wales	29	10	1	4	14
Victoria	5	0	3	2	0
Queensland, Western Australia, South Australia	4	0	4	0	0
*Rurality*					
Metropolitan	30	5	6	5	14
Regional centres	0	0	0	0	0
Medium/large rural towns	2	2	0	0	0
Small rural towns	6	3	2	1	0
*1-Item Literacy Screener—confidence filling out medical forms oneself*					
Not at all / A little bit	0	0	0	0	0
Somewhat	1	1	0	0	0
Quite a bit	10	4	1	3	2
Extremely	27	5	7	3	12
*Patient Clinical Information (self-reported)*					
**Type of cancer**					
Gynaecological	4	2	2		
Gastrointestinal	4	3	1		
Sarcoma/blood/soft tissue	3	3	0		
Lung	3	2	1		
Breast	1	0	1		
Other	3	0	3		
**Stage of cancer**					
Local	4	1	3		
Spread to lymph glands	1	1	0		
Spread to other body parts	12	7	5		
Not sure	1	1	0		
**Treatment**					
Surgery	14	7	7		
Immunotherapy	5	4	1		
Chemotherapy	13	9	4		
Radiation therapy	9	6	3		
Hormone tablets	1	0	1		
Targeted therapy	2	1	1		
*Professional Information*					
**Role**					
Research coordinator					7
Medical oncologist					3
Genetic counsellor					2
Geneticist					2
**Health systems**					
Public					6
Private					2
Both					6
**Confidence obtaining consent to research involving genomic testing**					
Not very confident					0
Confident					8
Very confident					6
Years working in oncology, M (SD) [Range]					11.7 (10.5) [1–29]

Data are number of participants (%), unless otherwise stated

*MoST:* Molecular Screening and Therapeutics program, * RCA:* Rare Cancers Australia, * HCPs:* healthcare or research professionals

### Initial QPL development

Round 1 interviews generated four categories of questions. Illustrative quotations are included below and in Table 3.

1. ***Questions about the Genomic Testing Process***.

Participants suggested the QPL should clarify the purpose of CGP, potential benefits of testing, and whom to contact with questions arising before/after enrolment. They emphasised the importance of clear expectations about what testing involved, including what patients needed to do, realistic timeframes for receiving results, and potential costs.

*“How do the results help me? What are they going to be useful for?”* (P17_MoST).

2. ***Questions about Receiving Results***.

Common questions related to the types of results patients could receive, who would deliver them, whether there would be enough time to discuss results, and whether plain language would be used. Participants wanted to know who could support them if they were distressed or confused by results; some wanted to know whether follow-up testing of their samples, or re-analysis of their data, may be done in the future as new treatments emerged.

*“[I’d like] some reassurance that… we’ll continue to look for more biomarkers as more research becomes available.”* (P35_MoST).

3. ***Questions about Potential Outcomes of Testing***.

Participants asked about treatments that may be offered after testing, and the likelihood of actionable results. All participant groups stressed the need to temper expectations through transparent discussions about the modest chance of individual clinical benefit. Many noted that while CGP inevitably raises hopes for effective personalised therapies, multiple barriers to treatment may persist even when an actionable target is identified. The QPL was therefore viewed as an opportunity to promote understanding and support patients and families in coping with uncertainty and possible disappointment.

*“Initially you’re excited to get an invitation to participate [in CGP] because there’s an opportunity there for some hope for a customised treatment. So*,* your thinking is maybe coloured a bit by that*,* and [my] family too…were excited initially. There’s that emotional layer that’s in there*,* and you’ve got to be realistic.”* (P17_MoST).

*“I think most [patients] go in with absolutely no idea… it’s just this wonderful appeal that they’re going to have this personalised medicine approach. So*,* I think you really have to tell them and educate them about the likely outcome.”* (P29_Oncologist).

Many participants wanted to understand whether results may have implications for family or insurance. As the purpose of the MoST program was tumour-focused CGP, a germline result (i.e., indicating a possible heritable variant) would be incidental and require confirmatory testing outside the program. HCPs emphasised the importance of clarifying this distinction. Nonetheless, participants felt that outlining potential positive or negative consequences for relatives was relevant to informed consent for CGP. Some suggested that patients may benefit from guidance around discussing relevant CGP results with relatives.

*“It would be really important to make sure that [patients] understand the difference in tumour testing versus germline testing so they don’t get those two confused. [CGP] is specifically for the management of their cancer.”* (P21_Geneticist).

*“What if [CGP results indicated] something negative? What are those negatives likely to be for my family? Because that could actually influence my consent… I won’t bother because that’s got too many consequences for my kids and grandkids.”* (P17_MoST).

4. ***Questions about the Genomic Study/Research Team***

Few participants spontaneously raised questions about the genomic research program or team, but when prompted they felt that knowing the institution/s involved was useful, as it helped patients judge the organisation’s credibility and the staff’s expertise. Some wanted to know about the program’s funding. Altruistic motives were commonly raised; patients wanted to know the purpose, scale and timeframe of the research, and how their participation could help others. Queries around privacy, security, storage and sharing of data were also raised, with some participants emphasising the importance of transparency.

*“[I’d want to know] what the overall [research] aim was – that if it doesn’t find anything for me*,* it’ll still contribute towards a general knowledge base – and whether it was just Australian based or an international project.”* (P25_RCA).

### Opinions about the Need for a QPL and Practical Considerations

As well as discussing specific questions that patients/families may wish to ask about genomic tests and research, participants (hypothetically) reflected on the value of a QPL in this context. Participants liked the idea of a prepared set of questions to consider, because this could help address gaps in understanding that patients might be unaware of, but they also acknowledged that some patient questions may be idiosyncratic. They felt a QPL could be empowering in an emotionally vulnerable situation and prompt clinicians to make space for patients’ and carers’ concerns and queries, thereby supporting them to become more fully informed without necessarily altering their testing decisions. One patient asserted that asking questions of clinicians could improve the care patients receive, and another mentioned searching the internet for suggested questions in anticipation of their diagnosis.

*“As a patient*,* you feel like you can’t query anything… So*,* I think something that’s formalised for questions you can ask*,* emotionally*,* that’s actually quite a helpful thing. It could also be good for the doctor to have the mindset that patients do have reasonable questions*,* and they need to allocate time to listen to those questions and explain.”* (P02_RCA).

*“I’m not saying it would have changed my consent*,* but it would have probably felt more comprehensive*,* what I knew about the process I was going into. So that would have been helpful.”* (P17_MoST).

Many participants preferred receiving a QPL several days before the consultation in which genomics participation would be discussed and/or finalised: *“People can read [the QPL] before their chat with the research coordinator*,* then in the appointment they can [seek] clarification… and then that will cement it because…you hear about the same thing in two different ways.”* (P21_Geneticist).

However, some HCPs involved in the MoST consent process were concerned about giving questions to patients without supplying answers. They therefore suggested including with the questions a prepared general answer to each one: *“I guess my worry is that they’d have a list of questions and start to think about the answers*,* that they could be potentially misinformed answers*,* as in*,* they’ll create an answer in their head and then that’s what they’ll believe and understand*,* versus knowing what the accurate answer is.”* (P27_Research staff).

**Table 3:** *Illustrative Participant Quotations from Study Interviews*

**Questions about the Process of Genomic Testing**
*“I think one of the things that I would ask [is] will it hurt? What does it involve? What are the specifics of doing this? … It’s not another operation*,* is it?”* (P15_Carer)
*“If you’ve done something and you’re a person that expects feedback a week later and then now it’s three weeks and four weeks*,* it can generate anxiety.”* (P17_MoST)
*“Usually*,* I do get questions about costs. So*,* they want to know if they have to pay anything to be part of the program or if their treatments later on*,* if they do match to a clinical trial*,* if there’s going to be expenses involved there.”* (P20_Research staff)
**Questions about Receiving Results**
*“That’s very important*,* the who will tell you*,* the how you will be told*,* and the format in which you will be told so you can understand.”* (P17_MoST)
*“[They should] be reminded… if you do need support*,* you’ve got these contacts*,* this is how you reach them. Because it can be so confusing and for a lot of these patients*,* they have a lot of medical staff already involved in their care. So*,* it can get quite confusing about who to contact and some of these people don’t have much time either. So… you don’t want them spending that time being stressed.”* (P20_Research staff)
**Questions about Potential Outcomes of Testing**
*“So*,* there’s two steps. You’ve got to find a biomarker and then you’ve got find a trial that’s open. There’s the chance that even if you find something*,* you might not find a trial that’s open… Even if you do find some druggable target*,* you might not be able to get the drug for the druggable target.”* (P12_Genetic counsellor)
*“I actually like to make sure that there’s no false hope given. I think that’s really important. So*,* letting them know*,* like I try to emphasise*,* there’s really no guarantee…”* (P27_Research staff)
*“The disappointment it wasn’t a match… I wasn’t prepared how to react. And I think that was probably important*,* is how I could help provide that support. It’s really hard when you’re trying to kind of temper a whole heap of emotions there.”* (P15_Carer)
*“This cohort of patients*,* they do understand that the testing that we’re doing may not necessarily benefit them in any way. So*,* I guess their concern tends to lie more on the impacts on their family…”* (P13_Research staff)
*“Being father of three sons*,* I thought if there’s something genetic here that I might be able to help them in the future.”* (P33_MoST)
*“Definitely [want to know] about consequences for insurance. I think that’s quite an emotive topic.”* (P07_RCA)
**Questions about the Genomic Study/Research Team**
*“I think the only thing that people really care about [is] that the credibility of the institution that’s running it is there*,* and that they will maintain their confidentiality.”* (P21_Geneticist)
*“I think probably one of the first questions I’d have is: will the information be shared with other parties?”* (P16_Carer)
**Opinions about Need for QPL and Practical Considerations**
*“My experience is that the better questions you ask a professional of any sort*,* the better engagement you get. The better questions you ask*,* the better care you get. So*,* I think a prompt list*,* for many people*,* would be helpful.”* (P03_MoST)
*“Even before I got my initial diagnosis and there was a possibility…that I did have cancer*,* I did Google ‘questions to ask your specialist’ in case.”* (P07_RCA)
*“I think a lot of people don’t know what questions to ask.”* (P35_MoST)
*“[It should have] blank spaces at the bottom of those questions for any weird and wonderful [questions] that are specific to your situation that you can throw to the doctor.”* (P24_RCA)
*“A lot of the time you can get in there and just totally forget to ask your question*,* and then you walk away and think ‘I should have asked that question.’ So yeah*,* that sounds like a good plan to get that kind of thing beforehand*,* so you know what’s happening when you go in.”* (P22_MoST)
*“I just really like the idea of preparing people’s minds before they come.”* (P08_MoST)
*“You may as well give them the list of questions with a list of answers already written down*,* like a Frequently Asked Questions…”* (P38_Research staff)
*“If an answer or a response was already underneath that question*,* it might alleviate any worry or stress to a degree.”* (P13_Research staff)

### QPL refinement

Following iterative research team discussions, participants’ proposed questions were organised into three categories: (i) Comprehensive Genomic Profiling, (ii) Results of CGP, and (iii) About the Genomic Research Study. In line with several participant recommendations and QPL templates, a fourth section titled ‘Add your own questions’ was included to encourage patients to write additional questions, if desired.

Although the original plan was to develop model answers as a separate resource to support consent staff discussions, interview feedback supported integrating answers directly into the QPL, creating a patient-facing question-and-answer (Q&A) tool. Answers were kept general and brief, covering the fundamental information while accurately reflecting CGP processes within the MoST program. Participants emphasised the importance of encouraging patients to seek personalised information from clinical/research professionals. Use of the SHeLL Health Literacy Editor led to extensive textual edits, including shorter sentences, reduced complexity, active-voice phrasing, and simpler vocabulary (where possible). Following revision, the question list was assessed at Grade 7.4 and the full Q&A at Grade 8.4 readability level.

#### Participant feedback

Overall, participants viewed the draft questions and answers positively, perceiving the QPL as valuable for patients considering participation in a CGP study.

**Structure**,** Content and Scope.** Participants endorsed the section order and *“liked the range of questions”* (RCA). The scope was highlighted as a strength, with comments that the tool had *“really good coverage”* (Carer), included *“very important questions to ask”* (MoST), and addressed *“everything I would have considered”* (MoST). Minor structural edits were suggested, such as combining related questions or splitting multi-part items. All participant groups re-emphasised the importance of explaining that a patient may still be unable to take part in a treatment trial even if a biomarker and relevant trial were found. One clinician suggested reassuring patients that their oncologist would continue providing the best available treatments, regardless of CGP outcomes. Additional suggested content included: why CGP testing takes time, and sources for further support (e.g., advocacy groups).

**Readability.** Participants from all groups found the wording easy to understand overall, valuing clear explanations of technical terms and avoidance of jargon. Patients commented that key concepts were *“explained beautifully”* (MoST) and that the QPL struck a *“good balance between not needing a degree to understand*,* as well as not dumbing it down”* (RCA). The *“simple and plain language”* (HCP) was perceived as appropriate for a range of audiences, including those with lower literacy or from culturally and linguistically diverse backgrounds; one participant compared it favourably with the MoST Participant Information Statement. Only minor wording changes were suggested.

**Utility and Implementation.** Patients and HCPs viewed the QPL as a helpful supplement to routine information. Patients felt *“empowered by”* the tool, with one saying it offered *“a bit of autonomy and a sense of control”* (RCA). Carers valued the resource giving *“permission for patients to ask questions*,* without premise or judgement”* and felt a prepared list *“takes stress off patients from having to come up with questions in a stressful setting.”* Participants favoured reviewing the QPL in advance so they could *“look over [it] with family”* (RCA) to prepare for discussion and decision-making. Its perceived value lay in helping *“arm patients with all the relevant information and means to receive more”* (HCP).

### Characteristics of final QPL

The final question list (Table 4) includes a brief explanatory introduction followed by 29 items in three categories: Comprehensive Genomic Profiling, Results of CGP, and About the Genomic Research Study, followed by an ‘Add Your Own Questions’ section. The complete Q&A is provided in the Supplementary Material. Since the MoST program has completed recruitment, answers were slightly adapted to reflect Omico’s Cancer Screening Program, which now offers CGP in a similar format to MoST.

**Table 4:** *Final CoGenT Question Prompt List*

Questions you could ask about comprehensive genomic profiling
Right now, doctors offer comprehensive genomic profiling mainly as part of medical research. But this kind of testing is becoming part of standard care more and more as time goes on.
*PART 1. * ***Comprehensive Genomic Profiling***
1. What is comprehensive genomic profiling?
2. How could this testing help me?
3. What does the testing involve? Will I need a new biopsy to get a sample of my tumour tissue for testing?
4. Will the testing hurt?
5. Will it cost me any money?
6. How long will it take to get my test results?
7. What should I do while I wait for my results?
*PART 2. * ***Results of Comprehensive Genomic Profiling***
1. Who will tell me my results, and how?
2. What kind of results could I get?
3. How likely is it that I will get a result that could guide my treatment?
4. If my results can guide treatment, will there be a treatment or trial suited to me? If there is, how will I join the trial?
5. What types of treatment could my report suggest, based on these results?
6. What if I don’t get a result that can guide treatment?
7. Who can help make sense of my results, answer my questions, or talk through my concerns about the results?
8. Will the research program test my sample again in the future? If there is a new treatment in the future, will someone get in touch with me?
9. What is a germline gene change?
10. Could my test results affect my job or my insurance?
11. Could my test results matter for my relatives?
12. Should I tell my relatives about my test results? Will someone help me do that?
13. What if I need support to cope with my feelings about the results? Who could give that kind of support to me or my relatives?
*PART 3. * ***About the genomic research study***
1. Why is the research team doing this study?
2. How might it help other people if I take part in this research?
3. What extra things will I need to do because I’m in a study?
4. Will someone explain the study to me in plain language that makes sense to me?
5. How can I find out more about the study, the team’s skills and knowledge, the organisations involved and their standing?
6. Where does the funding for this research come from?
7. How long has the study been going? When will it finish? How will I find out what the research findings are?
8. Who should I get in touch with if I have questions about the study now or later?
9. How will the research team store my details and results? Who will be able to see my results?

## Discussion

This study reports on the rigorous development of an evidence-based QPL, with accompanying answers, designed to support communication and decision-making for cancer patients considering taking part in a genomics study involving CGP. Patients, carers, clinicians and research staff directly shaped the tool’s scope, content, and Q&A format, and endorsed its value, acceptability and anticipated usefulness in guiding informed consent for genomics studies. The need for such a tool was demonstrated in a recent systematic review of interventions to support patient communication and decision-making about participation in health research, which identified limited peer-reviewed studies, with most interventions focused on treatment trials rather than testing-based research (Hersch et al. 2025).

Interview findings demonstrated that patient/carer information needs clustered around practical aspects of the testing process, types and implications of possible results, next steps, and the support available when navigating testing outcomes. Understanding research aims and the contribution of genomics studies to scientific progress was recognised as important for well-informed consent, although for some patients this was less salient than personal clinical implications of CGP. While the QPL seemed to cover common and important questions, participants emphasised the need of encouraging individuals to add their own questions alongside the structured resource.

The identified information needs closely align with published guidance on key aspects of the genomic consent process, such as the Australian National Model of Consent for Clinical Genomic Testing (NSW Ministry of Health 2021). Our findings reinforce that informed consent necessitates information presented in plain, accessible language, having sufficient time for deliberation, and opportunities to discuss concerns and ask questions. Participants highlighted the potential value of the QPL in signalling to health professionals that patients’ (and carers’) questions are appropriate and welcome. In addition to procedural and cost-related information, participants recommended including guidance on accessing professional services (e.g., genetic counselling or mental health) and peer support, consistent with the National Model of Consent (NSW Ministry of Health 2021).

The findings also resonate with recommendations that consent materials should prepare patients for the full spectrum of possible test results and implications, including unexpected or uninformative outcomes (NSW Ministry of Health 2021). This is particularly important given evidence of significant knowledge gaps and misconceptions about results interpretation among patients undergoing genetic testing (Nikitara et al. 2025). A recent systematic review (Salam et al. 2025) demonstrated that limited patient understanding of biomarkers, and how their testing relates to treatment, contributes to uncertainty, confusion, worry, and difficulties communicating with relatives. Our participants echoed the literature’s emphasis on managing patient expectations, particularly in light of the emotional burden associated with receiving disappointing results (Salam et al. 2025).

### Strengths, limitations and future directions

A major strength of this study is its rigorous co-design approach, involving diverse interest-holder groups with varied experience of genomic testing and research. Patient input was central to designing a QPL to meet patient needs and preferences, while involving clinicians and research staff ensured clinically relevant content (Ramlakhan et al. 2023). The rigorous, staged development process supported refinement of the content over multiple steps for iterative improvement. We detailed this process to show how interest-holder input shaped the final QPL and to address gaps in clarity and transparency noted in prior QPL literature (Ramlakhan et al. 2023).

The evidence-based answers accompanying the question list are broadly applicable across genomic testing and research contexts, or readily adaptable to specific genomic settings. These answers may be provided directly to patients and/or utilised by HCPs as a template for discussions, offering guidance on both content and phrasing. Previous research has shown providing answers alongside a QPL can significantly improve patients’ subjective understanding of the clinical topic (Ogawa et al. 2007). The use of the SHeLL Health Literacy Editor (Ayre et al. 2024) in the current study substantially improved QPL readability, addressing a known barrier to informed consent in genomic settings. The availability of prepared answers, including links to further resources, may also reassure HCPs who are concerned about addressing complex questions (Ramlakhan et al. 2023) or about patients/families seeking answers from unreliable sources. The answers are provided in the Supplementary Material as a model for adaptation for the needs of specific settings, although some content will require updating as relevant policy and legislation evolves (e.g., insurance implications of germline findings) (Tiller 2025).

The QPL is flexible in format and can be delivered digitally or in print. Within the current broader research program, the Q&A has been incorporated into the CoGenT dynamic consent platform – a novel online tool designed to support informed consent for CGP in research settings (Goncharov et al. 2022; Hersch et al. 2024). In this digital Q&A implementation, users initially view only the questions and select those of interest to reveal the corresponding answers. Early feedback suggests this ‘layered’ approach helps minimise cognitive overload while facilitating personal tailoring to individuals’ information preferences. We are currently exploring how the CoGenT resources could be implemented in practice, including which health professionals would use them and at which stage of the genomic oncology pathway (Alvaro et al., in preparation).

Several limitations should be acknowledged. Patient and carer participants were predominantly English-speaking, with relatively high levels of education and health literacy, and we did not collect ethnicity data. Future research should examine how to further enhance the QPL’s accessibility and utility among more diverse populations, particularly those with lower literacy, culturally and linguistically diverse groups, people living in regional or remote areas, and among a broader sample of carers/family members. This may include wording changes to further improve readability and/or exploring alternative formats of presenting information and delivery methods.

Real-world consent situations also differ from the hypothetical framing in this study. Further research is needed to evaluate the feasibility and effectiveness of this QPL in real-world contexts, including among patients at different disease stages making actual decisions about participating in CGP and/or genomics research. Such studies should incorporate validated measures of decision quality and psychosocial outcomes. The third section of the QPL is particularly pertinent when genomic testing occurs within a research context, as is often the case currently, and may be omitted when such testing is conducted within routine clinical practice.

## Conclusions

This is the first QPL specifically designed to support communication about decisions to undergo CGP and participate in research involving CGP, addressing key challenges in achieving informed consent in this complex setting. By combining a co-designed question list with evidence-based answers, this new consent resource fills an important gap in helping patients and families understand and navigate CGP. Participant feedback indicates high acceptability, perceived usefulness, and feasibility of the Q&A, with the potential to empower patients/families while supporting HCPs during consent discussions. Further research should evaluate the QPL in more diverse populations and assess its real-world effectiveness in improving informed consent and related outcomes among patients considering CGP and genomic research.

## Supplementary Information

Below is the link to the electronic supplementary material.

CoGenT question and answer list

## Data Availability

De-identified interview transcripts generated during the current study are available from the corresponding author on reasonable request.
