# Supplementary material for: Development of a question prompt list to support Consent for Genomic Testing (CoGenT) and research
Source: Hum Genet. 2026 May 21;145(1):44. doi: 10.1007/s00439-026-02839-0 (PMC13194191; doi:10.1007/s00439-026-02839-0)
Supplement: Supplementary file 1 — CoGenT question and answer list [file 439_2026_2839_MOESM1_ESM.docx]

Supplementary material to:

**Development of a question prompt list to support Consent for Genomic Testing (CoGenT) and research**

Journal: **Human Genetics**

**Authors:**

Jolyn Hersch^1,2^, Lauren O’Hara^1,2^, Phyllis Butow^1^, Mandy Ballinger^3^, Rebekah Laidsaar-Powell^1^, Nicci Bartley^1^, Kirsten McCaffery^2^, Anastasia Latin^1,2^, Christine Cockburn^4^, Caitlin Delaney^5^, Katherine M Tucker^6,7^, Ilona Juraskova^1^

^1^ The University of Sydney, Faculty of Science, School of Psychology, Psycho-Oncology Co-operative Research Group (PoCoG), Sydney, Australia

^2^ The University of Sydney, Faculty of Medicine and Health, School of Public Health, Sydney Health Literacy Lab, Sydney, Australia

^3^ University of New South Wales, Faculty of Medicine and Health, School of Biomedical Sciences, Centre for Molecular Oncology, Sydney, Australia

^4^ Rare Cancers Australia, Bowral, Australia

^5^ CareFully, Sydney, Australia

^6^ Prince of Wales Hospital, Nelune Comprehensive Cancer Centre, Hereditary Cancer Centre, Sydney, Australia

^7^ University of New South Wales, Faculty of Medicine and Health, School of Clinical Medicine, Sydney, Australia

**Corresponding Author:** Jolyn Hersch

ORCID: 0000-0001-5225-6639

[jolyn.hersch@sydney.edu.au](mailto:jolyn.hersch@sydney.edu.au)

**Questions you could ask about comprehensive genomic profiling**

Right now, doctors offer comprehensive genomic profiling mainly as part of medical research. But this kind of testing is becoming part of standard care more and more as time goes on.

We give general answers to the questions here. But you may also want to talk to your doctor or the research team.

**1. Comprehensive Genomic Profiling**

**1. What is comprehensive genomic profiling?**

Comprehensive genomic profiling means looking for changes in the genes or proteins in tumour tissue. The goal is to try and find changes in the tumour, called biomarkers.

Finding certain biomarkers in your tumour can suggest what type of treatment may be most useful for you.

**2. How could this testing help me?**

If this testing finds a useful biomarker in your tumour, you may be able to get a targeted therapy. This means a treatment that’s matched to the exact biomarker found in your tumour. Treatment might be through a research study, or your doctor might be able to arrange the treatment outside a study.

Please note that testing finds a useful biomarker in only 1 person out of every 3 people who have testing. This means that for most people (2 out of 3) testing does not find any biomarker.

**3. What does the testing involve? Will I need a new biopsy to get a sample of my tumour tissue for testing?**

When you had your tumour removed or tested before, the hospital or pathology service kept part of the tumour. The testing centre will get a small part of that tissue for the molecular profiling test. If there isn’t enough tumour tissue now, the testing centre could use a sample taken from you in the future.

**4. Will the testing hurt?**

The tumour testing doesn’t hurt because it uses tissue already removed from your body. You also need to give a blood sample. See *What does the testing involve? Will I need a new biopsy to get a sample of my tumour tissue for testing?*

**5. Will it cost me any money?**

You will not have to pay any money for the testing itself.

**6. How long will it take to get my test results?**

For most people, it takes 8-10 weeks from when you agree to testing until your cancer doctor gets the results. It takes this long because there are many steps to prepare for comprehensive genomic profiling.

**7. What should I do while I wait for my results?**

While you wait for the test results, follow the advice of the doctor who manages your cancer. The advice may be to carry on with your treatment, start new treatment, or not have treatment during this time.

The test results may not lead to any change in the treatment suggested for you.

Sometimes people feel anxious as they wait for their results and wonder what they’ll show. For advice about how to cope with feeling worried, see [Resources](https://cogent.org.au/resources).

**2. Results of Comprehensive Genomic Profiling**

**1. Who will tell me my results, and how?**

Your results will be in a report that goes to your cancer doctor.

Your doctor will tell you what the report says and what the results mean for you.

If you would like a copy of the report after seeing the doctor, ask the study team. See *Who should I get in touch with if I have questions about the study now or later?*

**2. What kind of results could I get?**

Your results report will describe the key tumour features found in the testing. This may tell your doctor something new about your illness, how it may progress, or what to do about it.

The main goal of comprehensive genomic profiling is trying to find a biomarker that can guide treatment of your cancer. If testing finds a useful biomarker, the report may suggest a matching treatment or clinical trial, if there is one. There might or might not be a treatment or clinical trial matching your biomarker right now. If there is not, the results may tell doctors something useful about how to treat your cancer in the future.

Sometimes, but not often, this testing may find a potential germline gene change that may run in the family. See *What is a germline gene change?*

**3. How likely is it that I will get a result that could guide my treatment?**

A result that can guide treatment means finding a biomarker that suggests what type of treatment may work for you. It is hard to predict whether the results of your test will be able to guide treatment or not.

Out of 100 people having tumour molecular profiling: about 33 get results that can guide treatment, and about 67 get results that cannot guide treatment.

**4. If my results can guide treatment, will there be a treatment or trial suited to me? If there is, how will I join the trial?**

If tumour testing finds a biomarker, your report may suggest that a certain treatment or clinical trial may suit you. A clinical trial is a study testing whether a treatment works and is safe for people with a certain illness.

Each trial has detailed rules about who can and cannot take part. For instance, it might depend on what treatments a person has had before. This means you might or might not be able to join a trial and get the treatment it offers.

Your cancer doctor will talk with you about what treatments you could have. If a trial is open to you, you can learn more about it to help decide whether to join it.

Taking part in any trial is up to you. You do not have to take part if you do not want to. You may wish to talk about this choice with your doctors and your loved ones.

If you do want to take part in the trial, your cancer doctor will explain how to join it.

**5. What types of treatment could my report suggest, based on these results?**

A targeted therapy is a drug treatment chosen for you based on the results from your tumour molecular profiling. Certain drugs may be good for you to try because they target and attack certain features found in your tumour. There are some drugs you need to get in hospital and others you can take at home.

Sometimes you can get this drug straight from your cancer doctor. But sometimes you can only get the drug through a clinical trial.

A trial treatment means research is checking in detail how well the drug works in your type of cancer. It’s important to note there is good science behind the drugs used in clinical trials.

**6. What if I don’t get a result that can guide treatment?**

In many cases, this testing will not find a useful biomarker that can guide treatment. When this happens, it is very normal to feel disappointed. Often though, people feel some peace of mind from knowing that they explored this possibility.

If testing doesn’t find a biomarker, your cancer doctor will keep seeing you and caring for you.

Your cancer doctor will look for other treatment options to help manage your cancer. Treatment could be through the cancer doctor or through a clinical trial where joining doesn’t depend on tumour profiling results.

**7. Who can help make sense of my results, answer my questions, or talk through my concerns about the results?**

The doctor who manages your cancer should be able to explain your results in plain language and answer your questions.

Based on your results, the doctor may suggest you talk to someone else. This could be a genetic counsellor or an expert in genomic medicine.

If you have questions about the research program itself, you can get in touch with the research staff. See *Who should I get in touch with if I have questions about the study now or later?*

**8. Will the research program test my sample again in the future? If there is a new treatment in the future, will someone get in touch with me?**

The program will not test your sample again, but the research team will re-analyse the study data over time.

In the future, there could be a new treatment that may suit you. If this happens, the research team will update your report and re-send it to your cancer doctor.

The doctor will then get in touch to talk with you about any new options that may suit you.

**9. What is a germline gene change?**

The main goal of tumour molecular profiling is trying to find a biomarker that can guide treatment of your cancer. But the test might also detect other potential gene changes called germline gene changes. A germline gene change can raise your chance of getting a certain illness, such as cancer. The gene change may also be present in your relatives.

Detecting a potential germline change is not common: it happens in about 15 of every 100 people tested. Further testing is needed to check if the germline gene change is real. It turns out to be real in about 5 out of 15 people who have further tests.

A germline gene change may run in the family, meaning that sometimes parents could pass it down to their children. If your relatives have the same gene change, they may be more likely to get a certain illness, like cancer.

The research team will tell you if there’s a potential germline finding.

**10. Could my test results affect my job or my insurance?**

If your report says tumour testing found a potential germline change (see *What is a germline gene change?*), you don’t have to tell anyone at your work. You may choose to have further testing to check if the germline change is real. If you confirm the germline change is real, and you later want to get some types of insurance, you may need to tell the insurer your germline testing result.

Getting private health insurance does not depend on your health, so no-one will ask you about your health or illness. But to get most life insurance products, you must answer questions about any illness in yourself or close relatives. These products offer cover for things like death, long term disability, trauma, and income protection. When you get life insurance, you must tell the insurer about your cancer. You only have to tell them about germline genetic test results if the cover is worth more than a certain amount. The amount depends on the type of cover. For more details about this, see [Life insurance products and genetic testing in Australia](https://www.genetics.edu.au/SitePages/Life-insurance-products-and-genetic-testing-in-Australia.aspx).

NOTE: This information is right in October 2025, but relevant laws are likely to change in the future.

**11. Could my test results matter for my relatives?**

Sometimes, but not often, this testing may find a potential germline gene change (see *What is a germline gene change?*). Further testing is needed to check if the germline gene change is real.

A germline gene change may run in the family, meaning that sometimes parents could pass it down to their children. If your relatives have the same gene change, they may be more likely to get a certain illness, like cancer.

**12. Should I tell my relatives about my test results? Will someone help me do that?**

Sometimes, but not often, this testing may find a potential germline gene change that may run in the family (see *What is a germline gene change?*). If your report says there may be a cancer gene change, the research team will invite you to a Family Cancer Clinic. You may need a blood test to confirm the result.

At the clinic, a doctor or genetic counsellor will explain what the result means for you and your relatives. As part of this genetic counselling, they will talk with you about the next steps you could take.

It’s up to you whether to tell your relatives about a germline result.

If your relatives have the same gene change, they may be more likely to get a certain illness, like cancer. There may be ways to lower the chance of getting that illness (like surgery, a drug, or a healthy lifestyle). If you tell your relatives about the gene change, they can decide whether to get themselves tested for it too. A Family Cancer Clinic can help you work out how to tell your relatives and what to say.

Genetic counselling is free in the public health system. You may need to pay for genetic counselling if you get it in the private system.

**13. What if I need support to cope with my feelings about the results? Who could give that kind of support to me or my relatives?**

Both your GP and your cancer doctor can support you. They can also suggest other ways for you and your family to get any extra support you may need. For instance, they can refer you to a mental health professional or a support group. If testing finds a gene change that may run in the family, a Family Cancer Clinic can also support you.

Sometimes people feel anxious as they wait for their results and wonder what they’ll show. For advice about how to cope with feeling worried, see [Resources](https://cogent.org.au/resources).

**3. About the genomic research study**

**1. Why is the research team doing this study?**

Genomic research projects test new ways to offer ‘personalised’ treatments for each person’s cancer.

The research looks at whether tumour molecular profiling changes people’s treatment and how well they do.

**2. How might it help other people if I take part in this research?**

By taking part in the research, you will be helping to move science forwards. This may help people to get better treatments for cancer in the future.

**3. What extra things will I need to do because I’m in a study?**

If you decide to join the study, you will fill out the Consent Form and give a blood sample.

The online forms will ask you about your health background and your family’s health background. They’ll also ask some questions about how you are feeling and how you are doing with day-to-day tasks.

During the study, the research team will get in touch every few months to ask questions about how you’re doing. Being in the study does not cost any money.

**4. Will someone explain the study to me in plain language that makes sense to me?**

Any study will involve a team of research staff whose job is to help people understand the study. They will be ready to answer any questions or address any concerns you may have (see *Who should I get in touch with if I have questions about the study now or later?*). You can talk to them when they first invite you to think about joining the study, and any time later.

If needed, research staff will arrange for a trained person to translate between English and the language you prefer speaking.

**5. How can I find out more about the study, the team’s skills and knowledge, the organisations involved and their standing?**

Genomic studies mostly involve a group of people working as a team. These people may work at universities and other research groups, such as the Australian Genomic Cancer Medicine Centre. Members of the team are experts in health research and certain parts of medicine and science, like cancer and genetics.

The Participant Information Sheet for each study outlines these details. Sometimes there’s a study website you can look at too. The website for the CaSP Program is <https://www.omico.com.au/our-programs/cancer-screening-program-casp/>.

**6. Where does the funding for this research come from?**

Research funding mainly comes from either the government or companies that make drugs. The Participant Information Sheet for each study says where the study’s funding comes from.

**7. How long has the study been going? When will it finish? How will I find out what the research findings are?**

To find out these details about a certain study, ask a member of the study team (see *Who should I get in touch with if I have questions about the study now or later?*).

The study team may send letters or emails sharing updates about how the study is going from year to year.

**8. Who should I get in touch with if I have questions about the study now or later?**

If you have questions about the research program, you can get in touch with the study team. To reach the CaSP Program team, email [casp@omico.org.au](mailto:casp@omico.org.au) or call 1800 954 350.

For any questions about your own health and care, the best people to ask are your cancer doctor and GP.

**9. How will the research team store my details and results? Who will be able to see my results?**

The research team will store all your details and results so they stay secure and private. The research team will not show your details to any other person unless you allow it.

Research programs use strong methods to keep your details secure, such as strict access controls, computer security, and staff training. The Participant Information Sheet describes this in more detail.

In the future, new research might start which could use your results to help move the science forwards even further. You can choose whether or not you let the research program share your results with future research projects. You do this as part of the consent form you sign when you agree to testing.
